# Treatment Options for Managing Anovulation in Women with PCOS: An Extensive Literature Review of Evidence-Based Recommendations for Future Directions

**DOI:** 10.3390/life15060863

**Published:** 2025-05-27

**Authors:** Alessia Mahoney, Arianna D’Angelo

**Affiliations:** School of Medicine, Cardiff University, Cardiff CF14 4YS, UK; dangeloa@cardiff.ac.uk

**Keywords:** fertility, PCOS, anovulation, clomiphene citrate, letrozole, myo-Inositol, ovulation, infertility, d-chiro-Inositol

## Abstract

Polycystic ovary syndrome (PCOS) is the expression of a complex alteration of the reproductive system. It is characterised by the increase in androgens, causing symptoms such as hirsutism, as well as infertility in many. This paper reviews whether Clomiphene Citrate, Letrozole and Inositol function better as monotherapy or combined therapy for anovulatory infertile PCOS patients. Ovulation and pregnancy rate were used as primary outcomes. PubMed and Scopus were the search engines of choice. Papers were excluded if patients were undertaking other fertility interventions, overlapping populations and non-RCT papers. It was found that co-prescribing Letrozole or Clomiphene Citrate alongside Metformin should be considered, Inositol should be examined as an alternative insulin sensitiser to Metformin and studies should be undertaken to identify the ideal dose and duration of Inositol therapy. Further large, well-designed, multi-centre studies should be conducted to solidify the claims of this review.

## 1. Introduction

PCOS is an endocrine disorder affecting 5–13% of women of reproductive age [1], with prevalence depending on which diagnostic criteria is used. The Rotterdam criteria is the most widely used, defined by the presence of two or more of the following: hyperandrogenism (HA), ovulatory dysfunction (OD) and polycystic ovarian morphology (PCOM). This gives rise to four PCOS phenotypes: phenotype-A (HA + OD + PCOM), phenotype-B (HA + OD), phenotype-C (HA + PCOM) and phenotype-D (OD + PCOM) [2].

The hormonal profile of PCOS typically includes elevated LH:FSH ratios, increased androgens (especially testosterone) and reduced sex hormone-binding globulin (SHBG), contributing to clinical manifestations such as hirsutism and ovulatory dysfunction. Notably, not all women with PCOS are anovulatory; phenotype C maintains ovulatory cycles despite hyperandrogenism and PCOM, underscoring the syndrome’s variability. At the ovarian level, hyperinsulinemia (due to insulin resistance) and intrinsic theca cell dysfunction exacerbate androgen production, impair folliculogenesis and contribute to infertility [3,4,5].

For ovulation induction, Clomiphene Citrate—a selective oestrogen receptor modulator—has traditionally been the first-line pharmacological treatment. Recent evidence, as reflected in the 2023 International Evidence-Based Guideline for the Assessment and Management of PCOS, supports Letrozole as the preferred first-line agent for ovulation induction due to its superior efficacy in achieving ovulation and live births compared to Clomiphene Citrate [6]. As an insulin-sensitising agent, Metformin is commonly used in PCOS to improve metabolic outcomes and may be beneficial for ovulation induction, particularly in women with insulin resistance or when used in combination with other treatments. Metformin is believed to stimulate ovulation in women with PCOS primarily by lowering insulin levels, which in turn may reduce androgen production by ovarian theca cells. This effect is thought to occur through downregulation of the steroidogenic enzyme CYP17, possibly via the PI3-K signalling pathway; however, the exact molecular mechanisms remain uncertain and somewhat conflicting in the literature [7], though this remains one of the most widely accepted explanations for how Metformin reduces hyperandrogenism and restores ovulatory function [8]. Reduced androgen levels help alleviate the inhibitory effect of androgens on granulosa cell function and aromatase activity, thereby improving oestrogen production and follicular maturation. Additionally, lowering androgens helps normalise the LH:FSH ratio and supports the selection of a dominant follicle, ultimately facilitating the resumption of regular ovulation in women with PCOS. Interestingly, Inositol (including stereoisomers myo-Inositol [MI] and D-chiro-Inositol [DCI]) has been newly discovered to be a potential second messenger regulating the activities of several hormones such as FSH, TSH and insulin [9,10].

This review tests whether CC, Letrozole and Inositol are more beneficial as monotherapy or combined therapy for patients with anovulatory PCOS. Primary outcomes of ovulation and pregnancy rate were chosen to assess efficacy. For strength of evidence, randomised-controlled trials (RCTs), systematic reviews and meta-analyses were selected.

## 2. Materials and Methods

**Figure 1:**
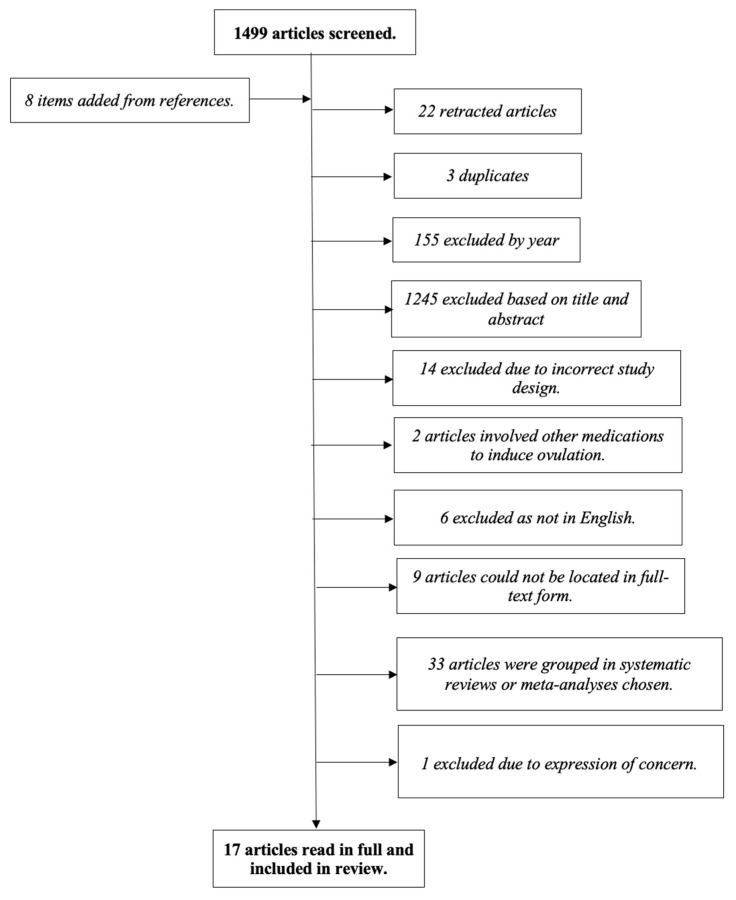
Screening process of the literature search.

The search strategy was inputted as seen in Appendix A. Study criteria involved anovulatory PCOS patients taking CC alone or in connection with Metformin OR Letrozole alone or in connection with Metformin OR MI alone or in connection with Letrozole, CC and Metformin. Exclusion criteria were patients undertaking other medical or surgical fertility interventions, as well as studies with overlapping populations. Further screening involved English-language papers with a publish date 2000–2024. The lower limit was chosen to capture research from the period leading up to the establishment of the Rotterdam criteria in 2003, which significantly influenced the diagnostic approach to PCOS. The upper limit of 2024 was selected to include the most recent and relevant evidence, including studies that reflect the latest updates in clinical guidelines, such as the 2023 International Evidence-Based Guideline for the Assessment and Management of PCOS. This ensures that the synthesis of evidence is aligned with current standards of care. The screening process is summarised in Figure 1.

Eight treatment combinations were compared in this study, as outlined below:

1. CC vs. Metformin;
2. CC vs. Metformin–CC;
3. Letrozole vs. Letrozole–Metformin;
4. Letrozole vs. CC;
5. DCI vs. placebo;
6. MI vs. Metformin;
7. MI vs. MI-DCI;
8. MI–Metformin vs. Metformin.

## 3. Results

A total of 17 studies were included in this analysis, conducted across 25 countries. These studies evaluated a range of interventions for PCOS, with primary outcomes of ovulation rate and pregnancy rate. Results across studies showed variation in effectiveness, with some interventions demonstrating improvements in ovulation but less clear effects on pregnancy rates. The comparisons with most clinical significance fell under the category of ‘Letrozole vs. CC’ and ‘DCI vs. Placebo’, favouring Letrozole and DCI, respectively. The following tables summarise the key findings from each study, with detailed comparisons of the medications evaluated, including Clomiphene Citrate (Table 1), Letrozole (Table 2), Metformin (Table 3) and Inositol (Table 4).

### 3.1. Clomiphene Citrate

A section to demonstrate the treatment combinations that include Clomiphene Citrate as a treatment option for women with infertility due to polycystic ovary syndrome.

**Table 1:** A table to summarise the literature included in this review regarding Clomiphene Citrate treatment options for women with infertility due to polycystic ovary syndrome.

Study Name	Dose	Study Design	Population	Ovulation Results	Ovulation Conclusion	Pregnancy Rate Results	Pregnancy Rate Conclusion
CC versus Letrozole							
*Legro, Letrozole* versus *Clomiphene for Infertility in the Polycystic Ovary Syndrome [11]*	CC: 50 mg daily Letrozole: 2.5 mg daily	Double-blind, multicenter trial	158 women total CC: 85 patients Letrozole: 73 patients	Significantly higher with Letrozole than with CC at each monthly visit (*p* < 0.01). Cumulative ovulation rate was higher with Letrozole than with CC (834 of 1352 treatment cycles [61.7%] vs. 688 of 1425 treatment cycles [48.3%], *p* < 0.001).			
*Abu-Zaid, Comparison of Letrozole and Clomiphene Citrate in Pregnancy Outcomes in Patients with Polycystic Ovary Syndrome: A Systematic Review and Meta-analysis [12]*		Meta analysis of RCTs	50 trials.	32 pooled trials found that ovulation rate was increased in Letrozole and CC groups by 75.42% and 62.45%, respectively. (RR: 1.20; 95% CI: 1.13, 1.26; *I*2 = 54.49%).	Letrozole intake leads to a higher rate of ovulation to CC.	Letrozole and CC on pregnancy rate was 33.15% and 22.84%, respectively across 44 trials. Letrozole intake significantly increases pregnancy rates by 44% compared to CC (RR: 1.44; 95% CI: 1.28, 1.62; *I*2 = 65.58%)	Letrozole intake leads to greater clinical pregnancy rates compared to CC.
CC vs. Metformin							
*Sharpe A, Morley LC, Tang T, Norman RJ, Balen AH. Metformin for ovulation induction (excluding gonadotrophins) in women with polycystic ovary syndrome. Cochrane Database Syst Rev. 2019 Dec 17;12(12):CD013505 [13]*		Systematic Review and Meta-Analysis	4 studies 2 with BMI < 30 kg/m^2^ 2 with BMI ≥ 30 kg/m^2^.	non-obese women: Metformin group has no clear difference in ovulation rates (OR 0.80, 95% CI 0.52 to 1.25; I2 = 0%; 5 studies, 352 women; low-quality evidence). obese women: Metformin may lower rates of ovulation (OR 0.29, 95% CI 0.20 to 0.43; I2 = 0%; 2 studies, 500 women; low-quality evidence).	Insufficient evidence to establish a difference in ovulation between Metformin and CC.		
*Misso ML, Costello MF, Garrubba M, Wong J, Hart R, Rombauts* L et al. *Metformin versus Clomiphene Citrate for infertility in non-obese women with polycystic ovary syndrome: a systematic review and meta-analysis. Hum Reprod Update. 2013 Feb;19(1):2–11 [14]*	Systematic Review and Meta-Analysis	4 RCTs.	Unable to detect a statistically significant difference between Metformin and CC.	Insufficient evidence to establish a difference between Metformin and CC in terms of ovulation.			
CC vs. Metformin–CC							
*Azargoon A, Fatemi HM, Mirmohammadkhani M, Darzi S. Is the Co-administration of Metformin and Clomiphene Superior to Induce Ovulation in Infertile Patients With Poly Cystic Ovary Syndrome and Confirmed Insulin-Resistance: A Double Blind Randomized Clinical Trial. J Fam Reprod Health. 2023 Mar;17(1):21–8 [15]*	Randomised Controlled Trial	151 women in this study. Group A: (Metformin +CC) = 76 subjects Group B: (placebo–CC) = 75 subjects (placebo–CC).	No remarkable differences in ovulation (*p* = 0.304) rates between Metformin and placebo. No significant differences in ovulation (*p* = 0.308) between the two groups.	No significant differences were detected in ovulation rate between the two groups.			
*Sharpe A, Morley LC, Tang T, Norman RJ, Balen AH. Metformin for ovulation induction (excluding gonadotrophins) in women with polycystic ovary syndrome. Cochrane Database Syst Rev. 2019 Dec 17;12(12):CD013505 [13]*	Systematic Review and Meta-Analysis	16 studies 7 with BMI < 30 kg/m^2^ 9 with BMI ≥ 30 kg/m^2^.	Combination of MT and CC was superior to the use of CC alone, with higher rates of ovulation (21 RCTs, 1568 women; OR 1.65, 95% CI 1.35 to 2.03; *I*2 = 63%; low-quality evidence).	Ovulation rates may be improved with Metformin and CC.			

Abbreviations: CC: Clomiphene Citrate; *p*: probability; RCTs: Randomised Controlled Trials; RR: Risk Ratio; CI: Confidence Interval; BMI: Body Mass Index; OR: Odds Ratio; MT: Metformin.

#### 3.1.1. CC vs. Metformin

There is insufficient evidence to establish a difference in ovulation between Metformin and CC among non-obese women (OR 0.80, 95% CI 0.52 to 1.25; I2 = 0%; 5 studies, 352 women; low-quality evidence) [13,14]. In obese women taking Metformin, there may be lower rates of ovulation (OR 0.29, 95% CI 0.20 to 0.43; I2 = 0%; 2 studies, 500 women; low-quality evidence). However, it should be stressed that the findings were inconsistent due to the high heterogeneity of the included RCTs, so should be interpreted with caution. Conclusions, therefore, cannot be confidently drawn, though CC may be better suited to obese patients.

#### 3.1.2. CC vs. Metformin–CC

A meta-analysis (Sharpe et al. 2019) collected 21 RCTs that included a total of 1568 women combined. They presented higher rates of ovulation when Metformin was combined with CC compared to the use of CC alone (OR 1.65, 95% CI 1.35 to 2.03; I2 = 63%; low-quality evidence), regardless of obesity status (*p* = 0.16). Since the publication of the aforementioned meta-analysis [13], a recent double-blind RCT showed no remarkable differences in ovulation rates between Metformin and placebo (*p* = 0.304), and no significant differences in ovulation (*p* = 0.308) between the two groups [15].

### 3.2. Letrozole

A section to demonstrate the treatment combinations that include Letrozole as a treatment option for women with infertility due to polycystic ovary syndrome.

**Table 2:** A table to summarise the literature included in this review regarding Letrozole treatment options for women with infertility due to polycystic ovary syndrome.

Study Name	Dose	Study Design	Population	Ovulation Results	Ovulation Conclusion	Pregnancy Rate Results	Pregnancy Rate Conclusion
Letrozole vs. CC							
	*(See Table 1)*						
Letrozole versus Letrozole–Metformin							
*Liu, Comparison of Clomiphene Citrate and Letrozole for ovulation induction in women with polycystic ovary syndrome: a prospective randomized trial [16]*	Letrozole: daily dose of 5 mg for 5 days (from day 3 to day 5 of the menstrual cycle) Metformin: daily dose of oral 1000–1500 mg	Prospective randomised trial	Letrozole = 62 patients Letrozole–Metformin = 57 patients	Ovulation rate (75.4% versus 71.5%) was higher in the group Letrozole–Metformin than in the Letrozole-alone group. But no significant difference (*p* > 0.05).	Letrozole +Metformin has slightly higher ovulation rate compared with Letrozole treatment.	The pregnancy rate (57.9% vs. 46.8%) were higher in the group Letrozole–Metformin than in the Letrozole alone group. But no significant difference (*p* > 0.05).	Letrozole +Metformin had slightly higher pregnancy rate (57.9% vs. 46.8%) compared with Letrozole treatment.

Abbreviations: CC: Clomiphene Citrate; *p*: probability.

#### 3.2.1. Letrozole vs. Letrozole–Metformin

Our search yielded one paper for this comparison [16]. When analysed, it showed the odds ratio as 1.27 (95% Cl 0.64 to 2.51; 134 women) [13]. The trial demonstrated that the ovulation rate (75.4% versus 71.5%) was higher in the group Letrozole–Metformin than in the Letrozole-alone group. Similarly, the pregnancy rate (57.9% vs. 46.8%) was higher in the group Letrozole–Metformin than in the Letrozole-alone group. But with both outcomes, no significant difference (*p* > 0.05) thus insufficient evidence of a beneficial effect. Regardless, Reproductive Medicine Guidelines [17] argue that there is fair evidence based on the well-designed trial to support Letrozole for ovulation induction (Grade B).

#### 3.2.2. Letrozole vs. Metformin

There are no head-to-head trials comparing the efficacy of Metformin to Letrozole alone. However, a double-blind, multicentre large RCT compared Letrozole with CC for ovulation induction. From this, Letrozole was superior (higher cumulative live births 27.5% vs. 19.1%, *p* = 0.007) [11]. Although CC is still the prevailing first-line therapy, the use of Letrozole for this population has increased. This was solidified by a 50-trial meta-analysis [12] that revealed a 20% higher rate of ovulation in the Letrozole group (RR: 1.20; 95% CI: 1.13, 1.26; I2 = 54.49%). Subgroup analysis revealed that the findings remained significant despite fewer than 4 years of infertility and with a BMI lower than 30 kg/m^2^. To dispute this, Roque et al. [18] did not find any significant differences in ovulation rate.

### 3.3. Metformin

A section to demonstrate the treatment combinations that include Metformin as a treatment option for women with infertility due to polycystic ovary syndrome.

**Table 3:** A table to summarise the literature included in this review regarding Metformin treatment options for women with infertility due to polycystic ovary syndrome.

Study Name	Dose	Study Design	Population	Ovulation Results	Ovulation Conclusion	Pregnancy Rate Results	Pregnancy Rate Conclusion
Metformin versus CC							
	*(See Table 1)*						
Metformin–CC versus CC							
	*(See Table 1)*						
Metformin–Letrozole versus Letrozole							
	*(See Table 2)*						
Metformin vs. MI							
*Misra, A randomised clinical trial comparing myoInositol and Metformin in PCOS [19]*	MI only = 1 g MI daily for 4 months MI–Metformin = 1 g MI + 1 g Metformin daily for 4 months 1 g Metformin daily for 4 months	Parallel 3-armed RCT (described as equivalence trial)	MI = 26 patients Age = 23.92 ± 3.70 years BMI = 24.63 ± 3.32 kg/m^2^ MI–Metformin = 22 patients Age = 21.9 ± 3.45 years BMI 25.02 ± 9.14 kg/m^2^ Metformin = 28 patients Age = 23.68 ± 4.23 years BMI = 25.44 ± 2.68 kg/m^2^			MI treated: 57.14% (*p* < 0.001) reported a pregnancy. Metformin treated: all patients reported a pregnancy (*p* < 0.001). (~Confounding factor: 5/9 had taken clomiphene for ovulation induction) MI–MetforminMetformin treated: all patients reported a pregnancy (*p* < 0.001).	
*Pourghasem, The effectiveness of Inositol and Metformin on infertile polycystic ovary syndrome women with resistant to Letrozole [20]*	4 g MI + 400 µg FA daily for 3 months Metformin 1.5 g + 200 μg daily200 µg FA daily for 3 months co-intervention: Letrozole 7.5 mg daily from third day of menstruation for 5 days in the third cycle	Parallel single-blind RCT3-armed RCT	MI + FA = 50 patients Age = 31.08 ± 3.31 years BMI = 29.79 ± 3.58 kg/m^2^ Metformin–FA = 50 patients Age = 31.06 ± 1.11 years BMI = 27.84 ± 3.68 kg/m^2^ FA alone = 50 patients Age = 30.42 ± 2.58 years BMI = 27.38 ± 4.02 kg/m^2^	No significant difference between the three groups (*p* > 0.05). Although the ovarian function is slightly lower in Letrozole –folic acid–Inositol than in Metformin+ folic acid–Letrozole groups.		Lower incidence of pregnancy in Letrozole–folic acid–MI group than other groups; however, it is not significant (*p* > 0.05).	
*Raffone, Insulin sensitiser agents alone and in co-treatment with r-FSH for ovulation induction in PCOS women [21]*	Intervention: MI 4 g MI + 400 µg FA daily for 6 months Control: 1500 mg Metformin daily (if no pregnancy occurred, intervention continued and FSH used for ovulation induction)	Parallel open-label RCT	MI = 60 patients Age = 29.1 ± 5.6 years BMI = 25 ± 2.1 kg/m^2^ Metformin = 60 patients Age = 29.7 ± 6 years BMI = 24.9 ± 2.7 kg/m^2^	CONTROL: 50% (30 of 60) of these patients restored spontaneous ovulation activity. In the patients with restored monthly menstruation, ovulation occurred after a mean 16.7 (+2.5) days from the first day of the menstrual cycle. INTERVEN: 65% of these patients restored spontaneous ovulation activity. Ovulation occurred after a mean of 14.8 (+1.8) days from the first day of the menstrual cycle.	Role of both Metformin and MI as first-line therapies for restoring a spontaneous ovulation in women with PCOS.	CONTROL: Pregnancy occurred spontaneously in 11 (18.3%) of these patients. The total pregnancy rate was 36.6% (22 women of 60). INTERVEN: Pregnancy occurred spontaneously in 18 (30%) of these patients. The total pregnancy rate was 48.4%, six of the 29 pregnancies (20.6%) evolved in spontaneous abortion.	Higher rate of pregnancies (48.3% vs. 36.6%) in the group treated with MYO, even if not statistically significant.
Metformin versus MI–Metformin							
*Misra, A randomised clinical trial comparing myoInositol and Metformin in PCOS [19]*	Inter: MI only = 1 g MI daily for 4 months MI–Metformin = 1 g MI + 1 g Metformin daily for 4 months Control: 1 g Metformin daily for 4 months	Parallel 3-armed RCT (described as equivalence trial)	MI = 26 patients Age = 23.92 ± 3.70 years BMI = 24.63 ± 3.32 kg/m^2^ MI–Metformin = 22 patients Age = 21.9 ± 3.45 years BMI 25.02 ± 9.14 kg/m^2^ Metformin = 28 patients Age = 23.68 ± 4.23 years BMI = 25.44 ± 2.68 kg/m^2^			MI: 57.14% (*p* < 0.001) of patients reported a pregnancy. Metformin group: all patients reported a pregnancy (*p* < 0.001). (~Confounding factor: 5/9 had taken clomiphene for ovulation induction) Both drugs: all patients with reported a pregnancy (*p* < 0.001).	
Metformin versus MI-DCI							
*Nordio, The combined therapy with myo-Inositol and D-chiro-Inositol reduces the risk of metabolic disease in PCOS overweight patients compared to myo-Inositol supplementation alone [22]*	MI group: 2 g of MI in powder MI + DCI group (40:1): 550 mg of MI plus 13.8 mg of DCI in soft gel capsule twice a day.	RCT	50 women with PCOS (BMI > 27 kg/m^2^, mean age 28 years old, range 18–41) MI group, 24 women MI + DCI group, 26 women	Improvement of the ovulation function and all the women ovulated after treatment (exact figures not seen).			

Abbreviations: CC: Clomiphene Citrate; p: probability; MI: Myoinositol; RCT: Randomised Controlled Trial; BMI: Body Mass Index; FA: Folic Acid; DCI: D-chiro-inositol.

#### 3.3.1. Metformin vs. MI

A parallel three-armed RCT by Misra described that the pregnancy rate was higher in women with PCOS who had taken Metformin compared to those who had taken MI [19]. However, the actual pregnancy rate in the Metformin group could not be fully assessed given the confounding factor of CC administration. When taken alone, MI yielded 57.14% of patients (*p* < 0.001) reporting a pregnancy. Pourghasem et al. utilised folic acid as their control (since it is commonly co-prescribed during pregnancy as a baseline) [20]. They found no significant difference between the interventions for ovulation and pregnancy rates (*p*  >  0.05). The previous papers are conflicting with Raffone et al. who found that 39/60 patients restored monthly ovulation in the MI group and 30/60 in Metformin [21]. The number of pregnancies in those with restored ovulation was 18/39 (46.1%) in MI and 11/30 (36.6%) in the Metformin group. This yielded an RR of 1.64 (95% CI 0.85–3.16) [23].

#### 3.3.2. Metformin Versus MI-DCI

Nordio and Proietti compared MI-DCI (in the 40:1 ratio) to Metformin [22]. Unlike other studies in this review, ovulation was confirmed when the level of progesterone was over 10 ng/mL, unlike the usual cutoff of 8 ng/mL. Although the paper reported improvement of the ovulation function and ovulation of all the women, exact values could not be located.

#### 3.3.3. Metformin Versus MI–Metformin

Earlier in this paper, it was discussed that Misra et al. observed better efficacy of Metformin compared to MI for pregnancy rate (57.14% vs. 100%) [19]. Interestingly, in those treated with both medications, all patients also reported a pregnancy (*p* < 0.001). This suggests that their co-prescription more effective than MI monotherapy.

### 3.4. Inositol

A section to demonstrate the treatment combinations that include Inositol as a treatment option for women with infertility due to polycystic ovary syndrome.

**Table 4:** A table to summarise the literature included in this review regarding Inositol treatment options for women with infertility due to polycystic ovary syndrome.

Study Name	Dose	Study Design	Population	Ovulation Results	Ovulation Conclusion
MI versus Metformin					
	*(See Table 3)*				
MI–Metformin versus Metformin					
	*(See Table 3)*				
MI-DCI vs. Metformin					
	*(See Table 3)*				
DCI vs. Placebo					
*Nestler JE, Jakubowicz DJ, Reamer P, Gunn RD, Allan G. Ovulatory and metabolic effects of D-chiro-Inositol in the polycystic ovary syndrome. N Engl J Med. 1999;340(17):1314–1320. [24]*	1200 mg DCI once daily	Parallel double-blind RCT	44 participants DCI = 22 patients Age = 29 ± 6 years BMI = 31.3 ± 2.4 kg/m^2^ Placebo = 22 patients Age = 26 ± 5 years BMI = 31 ± 2.2 kg/m^2^	Exceeded 8 ng per millilitre. 19/22 women in the DCI group (86 percent) ovulated during treatment. Only 6/22 women (27 percent) in the placebo group (*p* < 0.001).	We conclude that DCI improves ovulatory function.
*Iuorno MJ, Jakubowicz DJ, Baillargeon* JP et al. *Effects of d-chiro-Inositol in lean women with the polycystic ovary syndrome. Endocr Pract. 2002;8(6):417–423. [25]*	600 mg DCI daily	Randomised double-blind RCT	20 participants DCI = 10 patients Age = 28.2 ± 1.5 years BMI = 22.4 ± 0.3 kg/m^2^ Placebo = 10 patients Age = 26.5 ± 1.4 years BMI = 22.1 ± 0.3 kg/m^2^	(Progesterone > 8 ng/mL) 6/10 women (60%) in the DCI group ovulated in comparison with 2/10 women (20%) in the placebo group (*p* = 0.17).	DCI improves ovulatory function.
*Gerli, Effects of Inositol on ovarian function and metabolic factors in women with PCOS: a randomized double blind placebo-controlled trial [26]*	100 mg twice daily	Randomised double blind placebo-controlled trial	283 patients were randomised in two groups, receiving either Inositol or placebo.	8/136 Inositol-treated patients failed to ovulate. 17/147 placebo-treated patients failed to ovulate. statistically significant difference (Fisher’s exact test; *p* = 0.04; odds ratio, 0.38).	The Inositol-treated group had a significantly increased frequency of ovulation compared with the placebo group.

Abbreviations: MI: Myoinositol; MI-DCI: Myoinositol and D-chiro-inositol; DCI: D-chiro-inositol; RCT: Randomised Controlled Trial; BMI: Body Mass Index; *p* = probability.

#### DCI vs. Placebo

For this comparison, only the ovulation outcome was reported on. The greatest dosage of DCI used was 1.2 g [24]. Their study found that the frequency of ovulation was greater in the DCI group compared to placebo (86% vs. 27%) [24]. Iuorno et al. used half of the dosage of DCI compared to Nestler John E. et al. Although their sample size was smaller, ovulation was still superior in the intervention group compared to control (60% vs. 20%) [25]. Gerli et al. administered the lowest dosage of DCI of 100 mg twice a day [26]. Unlike the aforementioned studies, this RCT sample size was larger at 283 patients. Here, a statistically significant difference was seen between patients failing to ovulate in the DCI vs. placebo group (8/136 vs. 17/147) (Fisher’s exact test; *p* = 0.04; odds ratio, 0.38). This article hypothesises that DCI improves ovulatory function. However, the three studies had a small sample size and utilised different doses of DCI. Therefore, the risk of imprecision is present, and thus the findings are categorised as low certainty.

## 4. Discussion

This study provides a comparative evaluation of Clomiphene Citrate, Letrozole and Inositol in the management of infertility in women with polycystic ovary syndrome (PCOS). It adds to the evolving discourse on evidence-based, individualised reproductive care amidst a nuanced landscape where each treatment presents distinct advantages and limitations.

Letrozole emerged as the most effective treatment in terms of ovulation rate when compared to Clomid. This conclusion was present even in patients with fewer than 4 years of infertility and with a BMI lower than 30 kg/m^2^. This aligns with an expanding body of evidence that supports its use as a first-line therapy [6]. Its mechanism of action as an aromatase inhibitor prevents oestrogen production, leading to enhanced follicular development by promoting a favourable gonadotropin environment. Unlike Clomid, Letrozole does not exert anti-estrogenic effects on the endometrium or cervical mucus [27,28], which may account for improved pregnancy outcomes reported in various studies [11]. In this study, Letrozole’s superiority was evident in that it significantly increases pregnancy rates by 44% compared to CC [12], further validating its therapeutic value. Despite the clear benefits, there is still debate regarding the safety profile of Letrozole during pregnancy, despite its short half-life [29]. From our search, ovulation and pregnancy rates were maximised when Metformin was added to the Letrozole therapy, likely due to its impact on insulin resistance. Letrozole’s anti-estrogenic effects lead to a positive feedback loop on the pituitary gland to release more FSH, thus causing regular growth and maturation of follicles. Should patients have insulin resistance, these follicles would differentiate prematurely despite their ongoing optimised maturation by Letrozole. Thus, dual therapy allows for both maturation and ovulation at the appropriate time.

Clomid, though historically the first-line agent for ovulation induction, showed comparatively lower efficacy than Letrozole in terms of ovulation rate. However, Clomid remains a widely used option due to its affordability, accessibility and well-established safety profile. It was noted in this study that for the subset of obese patients, Clomid did yield positive outcomes in terms of ovulation rate [13], indicating that it may still be an appropriate option for certain patient subsets. However, due to heterogeneity, the conclusions should be drawn with caution. This variability in treatment response across studies in the meta-analysis may be due to differences in study populations, insulin resistance status and treatment duration. When considering dual therapy with Metformin, implementing Metformin was more beneficial than prescribing CC alone, especially for the subgroup of obese patients. This is believed to be due to the impact of insulin resistance on fertility [3,30]. This resistance can lead to compensatory hyperinsulinemia. Consequently, the high insulin levels can induce an early response to luteinising hormones on granulosa cells of small follicles, thus causing premature differentiation and anovulation. Therefore, although its selective oestrogen receptor antagonistic mechanism may result in promoting ovarian follicle development, without addressing insulin insensitivity, the follicles are not effectively utilised per cycle. Therefore, for those on Clomid therapy, co-prescription with Metformin should be considered. This dual therapy would be best suited particularly to patients with a BMI of over 30. Given the high heterogeneity of included RCTs, this recommendation should be considered with caution.

Inositol presented an interesting therapeutic profile in its main forms d-chiro-Inositol and myo-Inositol. It shows potential to improve ovulation as well as pregnancy rate, though the results were conflicted depending on the treatment comparison. Similarly to Metformin, MI facilitates the action of insulin [31]. Inositol acts as a secondary messenger in the insulin signalling pathway. When converted into Inositol triphosphate, insulin’s action is facilitated thus reducing the chance of compensatory hyperinsulinemia. In the three RCT studies that reported on DCI vs. placebo [24,25,26], each intervened with different daily doses of DCI: 1200 mg, 600 mg and 200 mg. Despite this, all three reported greater rates of ovulation compared to placebo with Folic Acid. This implies that DCI has beneficial effects regardless of dosage, but that studies to quantify the ideal dosage should be conducted. However, of the three studies reviewed, only one reported statistically significant results [26]. Notably, this study also had the largest sample size, suggesting that statistical power may have played a role in detecting meaningful effects. While the other two studies reported beneficial outcomes [24,25], these did not reach statistical significance. It is plausible that with larger sample sizes, these effects may have achieved significance as well. The evidence on myo-Inositol versus Metformin was conflicting. Notably, the trial that reported a positive benefit in ovulation rate [21] had a longer treatment duration of six months, while the studies with shorter durations of three [20] and four months [19] found no significant differences (for ovulation rate or pregnancy rate) [20] or concluded that the intervention was not superior (for pregnancy rate) [19]. This raises the possibility that the length of treatment may play a crucial role in the observed effectiveness, with longer durations potentially allowing more time for measurable improvements.

This study is not without limitations. The sample sizes of the papers were modest, which may limit statistical power and the ability to detect subtle differences between groups. The study designs did not include long-term follow-up, which prevents an evaluation of long-term effects on future pregnancies. Relying on systematic reviews and meta-analyses improved rigour but may have limited the detail of some comparisons. These limitations highlight the need for larger, multicentre studies with more robust design parameters and longer-term follow-up.

From the search, the most scope for further research was surrounding Inositol. The evidence is unclear as to whether MI is more effective than Metformin. The studies with the longest intervention period reported the most positive outcomes for MI. This suggests that extended treatment may be necessary for the therapeutic effects of MI to become fully apparent, and future trials could consider evaluating longer-term administration to better understand the time-dependent nature of responses. Another treatment comparison hinges on Inositol and Metformin both working as insulin sensitisers. Higher rates of ovulation were observed when Metformin was co-prescribed to patients taking CC or Letrozole monotherapy. That being so, an interesting topic of study might be to compare whether Inositol (MI or DCI) is more effective than Metformin as a co-prescription.

The findings from this review suggest that treatment with DCI is more effective than no intervention, highlighting its potential clinical value. However, as the included RCT studies reported varying outcomes potentially influenced by differences in dosage, further research is warranted to determine the optimal dosing strategy for maximising therapeutic benefit.

Some studies used the d-chiro-Inositol form of Inositol, while others used myo-Inositol, making direct comparisons difficult. Future research should aim to include both forms within the same study to better understand their relative and combined effects.

Of the treatment regimes, we identified that no RCTs compared the following: MI vs. placebo, DCI vs. Metformin, DCI/MI vs. Letrozole (±DCI/MI), DCI/MI vs. CC (±DCI/MI) and Letrozole vs. Metformin. Of note, although a trial comparing MI-DCI to Metformin has been conducted, results are unavailable, so further research is needed to determine its true effectiveness. Currently, there is no ideal dosage to draw effective conclusions.

Moving forward, research should continue to investigate the pathophysiology of these processes, particularly in diverse patient populations. Though subgroup analyses have tended to investigate obesity status, epidemiological analysis in a multi-centre, multi-city study might be beneficial. Additionally, long-term studies are warranted to evaluate the sustainability of treatment effects and patient adherence.

## 5. Conclusions

Our review has systematically provided a comprehensive up-to-date summary of the most reliable treatments for infertility in PCOS. We demonstrate that both Letrozole and Clomid consistently yield better outcomes when co-prescribed with Metformin, suggesting that Metformin should be considered as a concurrent therapy rather than solely a subsequent option. This approach may enhance treatment efficacy and should be further explored in clinical practice and future studies. In recent years, new developments have included the use of Inositol. These authors suggest considering Inositol as an alternative insulin-sensitiser to Metformin. However, ideal doses and treatment duration would need to be determined for both forms of Inositol (DCI and MI). Through the analysis, personalised therapeutic approaches that promote ovulation and pregnancy rates can be streamlined.

## Appendix A

***Clomiphene***

A PubMed and Scopus Search was performed to encompass papers from 2000 to 2024. The following search strategy was used: (Anovulatory OR infertility OR infertile) AND (“PCOS” OR “polycystic ovary syndrome” OR “polycystic ovarian syndrome”) AND (((“Clomiphene Citrate” OR Clomid OR clomiphene OR Clomifene OR Clomifène OR Chloramiphene OR “Clomifene citrate” OR Serophene) OR (Metformin OR Glucophage OR Glumetza OR “Glucophage XR” OR Fortamet OR Riomet)) OR ((“Clomiphene Citrate” OR Clomid OR clomiphene OR Clomifene OR Clomifène OR Chloramiphene OR “Clomifene citrate” OR Serophene) AND (Metformin OR Glucophage OR Glumetza OR “Glucophage XR” OR Fortamet OR Riomet))) AND (“randomized controlled trial” [pt] OR “RCT” OR “controlled clinical trial” [pt] OR randomized [tiab] OR placebo[tiab] OR “drug therapy”[sh] OR randomly[tiab] OR trial[tiab] OR groups[tiab]) AND (ovulation OR (pregnant OR pregnancy))

***Letrozole***

A PubMed and Scopus Search was performed to encompass papers from 2000 to 2024. The following search strategy was used: (Anovulatory OR infertility OR infertile) AND (“PCOS” OR “polycystic ovary syndrome” OR “polycystic ovarian syndrome”) AND (((Letrozole OR Femara OR “aromatase inhibitor”) OR (Metformin OR Glucophage OR Glumetza OR “Glucophage XR” OR Fortamet OR Riomet)) OR ((Letrozole OR Femara OR “aromatase inhibitor”) AND (Metformin OR Glucophage OR Glumetza OR “Glucophage XR” OR Fortamet OR Riomet))) AND (“randomized controlled trial” [pt] OR “RCT” OR “controlled clinical trial” [pt] OR randomized [tiab] OR placebo[tiab] OR “drug therapy”[sh] OR randomly[tiab] OR trial[tiab] OR groups[tiab]) AND (ovulation OR (pregnant OR pregnancy))

***Inositol***

A PubMed and Scopus Search was performed to encompass papers from 2000 to 2024. The following search strategy was used: (Anovulatory OR infertility OR infertile) AND (“PCOS” OR “polycystic ovary syndrome” OR “polycystic ovarian syndrome”) AND ((“Myo-Inositol” OR Inositol OR MyoInositol OR “D-chiro-Inositol”) OR ((“Myo-Inositol” OR Inositol OR MyoInositol OR “D-chiro-Inositol”) AND ((Metformin OR Glucophage OR Glumetza OR “Glucophage XR” OR Fortamet OR Riomet) OR (“Clomiphene Citrate” OR Clomid OR clomiphene OR Clomifene OR Clomifène OR Chloramiphene OR “Clomifene citrate” OR Serophene) OR (Letrozole OR Femara)))) AND (“randomized controlled trial” [pt] OR “RCT” OR “controlled clinical trial” [pt] OR randomized [tiab] OR placebo[tiab] OR “drug therapy”[sh] OR randomly[tiab] OR trial[tiab] OR groups[tiab]) AND (ovulation OR (pregnant OR pregnancy))
